# Supplementary material for: Entanglement Rényi negativity of interacting fermions from quantum Monte Carlo simulations
Source: Nat Commun. 2025 Mar 17;16:2637. doi: 10.1038/s41467-025-57971-8 (PMC11914579; doi:10.1038/s41467-025-57971-8)
Supplement: Supplementary file 1 — Supplementary Information [file 41467_2025_57971_MOESM1_ESM.pdf]

Supplementary Information for
"Entanglement Rényi negativity of interacting fermions from quantum Monte Carlo simulations"

Fo-Hong Wang and Xiao Yan Xu*

*Corresponding author. Email: xiaoyanxu@sjtu.edu.cn

This PDF file includes:

- Supplementary Note 1: Fermionic partial transpose in different representations
- Supplementary Note 2: Determinant Quantum Monte Carlo Methods
- Supplementary Note 3: DQMC implementation of fermionic partial transpose
- Supplementary Note 4: Additional results of the Rényi negativity
- Supplementary Note 5: Sign problem of Grover determinant
- Supplementary Figs. 1 and 2

Supplementary Note 1: Fermionic partial transpose in different representations

In this section, we briefly review the definition of fermionic partial transpose, which does not follow the original definition [1] extensively used in bosonic systems. Consider a lattice model described by complex fermion operators c_j and $c_j^\dagger$ satisfying anticommunication relations $\{c_j, c_k^\dagger\} = \delta_{jk}$, where $j, k = 1, \ldots, N$ are labels of sites (for simplicity we omit the index for internal degree of freedom). For convenience, we also introduce the Majorana basis, denoted as $\gamma_{2j-1} = c_j + c_j^\dagger$ and $\gamma_{2j} = -\mathrm{i}(c_j - c_j^\dagger)$. Under a bipartite scheme that divides the total system as $A = A_1 \cup A_2$, the fermionic partial transpose with respect to subsystem A_2, denoted by $\mathcal{O}^{T_2^f}$ with $\mathcal{O}$ as an operator (like the density operator ρ or just a single basis operator $|\{e_j\}\rangle\langle\{\bar{e}_j\}|$), is first defined in the coherent basis as [2]

$$U_{A_2}\left(|\{\xi_j\}_{j\in A_1}, \{\xi_j\}_{j\in A_2}\rangle\langle\{\bar{\chi}_j\}_{j\in A_1}, \{\bar{\chi}_j\}_{j\in A_2}|\right)^{T_2^f} U_{A_2}^\dagger = |\{\xi_j\}_{j\in A_1}, \{-\mathrm{i}\bar{\chi}_j\}_{j\in A_2}\rangle\langle\{\bar{\chi}_j\}_{j\in A_1}, \{-\mathrm{i}\xi_j\}_{j\in A_2}|, \tag{1}$$

where $|\{\xi_j\}\rangle = e^{-\sum_j \xi_j c_j^\dagger}|0\rangle$ and $\langle\{\bar{\chi}_j\}| = \langle 0|e^{-\sum_j c_j \bar{\chi}_j}$ are the fermion coherent states, and $U_{A_2} \equiv \prod_{j\in A_2}\gamma_{2j-1}$ is the partial particle-hole transformation which only turns the particles (holes) in the subsystem A_2 into holes (particles). After choosing an appropriate ordering such that $A_2 = \{N_1 + 1, \ldots, N\}$, one obtains fermionic partial transpose in the occupation number basis by expressing the coherent states in Supplementary Eq. (1) in terms of Fock states [2, 3]

$$\left(|\{n_j\}_{j\in A_1}, \{n_j\}_{j\in A_2}\rangle\langle\{\bar{n}_j\}_{j\in A_1}, \{\bar{n}_j\}_{j\in A_2}|\right)^{T_2^f} = (-1)^{\phi(\{n_j\},\{\bar{n}_j\})}|\{n_j\}_{j\in A_1}, \{\bar{n}_j\}_{j\in A_2}\rangle\langle\{\bar{n}_j\}_{j\in A_1}, \{n_j\}_{j\in A_2}|, \tag{2}$$

which is similar to the conventional partial transpose up to an additional phase factor

$$\phi\left(\{n_j\}, \{\bar{n}_j\}\right) = \left[(\tau_2 + \bar{\tau}_2) \bmod 2\right]/2 + (\tau_1 + \bar{\tau}_1)(\tau_2 + \bar{\tau}_2) \tag{3}$$

with $\tau_b = \sum_{j\in A_b} n_j$ the number of particles in subsystem A_b $(b = 1, 2)$. The definition in Supplementary Eq. (2) has been employed in the exact diagonalization calculations in Fig. 1 of the main text. Moreover, utilizing this definition, it is straightforward to show that

$$\begin{aligned}\mathrm{Tr}\left[\left(\rho^{T_2^f}\right)^k\right] = &\sum_{\{n_j^{(1)}\}\cdots\{n_j^{(k)}\}} \left\langle\left\{n_{j\in A_1}^{(1)}, n_{j\in A_2}^{(3)}\right\}\middle|\rho\middle|\left\{n_j^{(2)}\right\}\right\rangle\left\langle\left\{n_{j\in A_1}^{(2)}, n_{j\in A_2}^{(4)}\right\}\middle|\rho\middle|\left\{n_j^{(3)}\right\}\right\rangle\cdots\left\langle\left\{n_{j\in A_1}^{(k)}, n_{j\in A_2}^{(2)}\right\}\middle|\rho\middle|\left\{n_j^{(1)}\right\}\right\rangle \\ &\times(-1)^{\phi\left(\{n_j^{(1)}\},\{n_j^{(2)}\}\right)}(-1)^{\phi\left(\{n_j^{(2)}\},\{n_j^{(3)}\}\right)}\cdots(-1)^{\phi\left(\{n_j^{(k)}\},\{n_j^{(1)}\}\right)}.\end{aligned} \tag{4}$$

Specifically, note that $(-1)^{\phi(\{n_j\},\{n_j\})} = 1$ and $\phi(\{n_j\}, \{\bar{n}_j\}) = \phi(\{\bar{n}_j\}, \{n_j\})$, one can deduce that $\mathrm{Tr}[\rho^{T_2^f}] = \mathrm{Tr}[\rho] = 1$ while $\mathrm{Tr}[(\rho^{T_2^f})^2] = \mathrm{Tr}[(\rho\hat{X}_2(\pi))^2]$ with $\hat{X}_2(\theta) = e^{\mathrm{i}\theta\sum_{j\in A_2}\hat{n}_j}$ being the disorder operator and $\hat{n}_j = c_j^\dagger c_j$ the local density operator [4, 5].

In general, density operators can be written as a restricted superposition of products of Majorana operators. Assume that there are k (l) sites in subsystem A_1 (A_2), in which the Majorana indices are denoted by $\{m_1, \dots, m_{2k}\}$ ($\{n_1, \dots, n_{2l}\}$), a density operator can be expressed as [6, 7]

$$\rho = \sum_{\substack{\kappa,\tau, \\ |\kappa|+|\tau|=\text{even}}} w_{\kappa,\tau} \gamma_{m_1}^{\kappa_1} \cdots \gamma_{m_{2k}}^{\kappa_{2k}} \gamma_{n_1}^{\tau_1} \cdots \gamma_{n_{2l}}^{\tau_{2l}} \tag{5}$$

where $\kappa = (\kappa_1, \cdots, \kappa_{2k})$ and $\tau = (\tau_1, \cdots, \tau_{2l})$ represent various Majorana configurations. Here, κ_i and τ_j are the occupations of single Majorana modes, and $|\kappa| = \sum_j \kappa_j$ or $|\tau| = \sum_j \tau_j$ is the total number of Majorana fermions in the corresponding subsystem. We note that $w_{\kappa,\tau} \neq 0$ only if $|\kappa| + |\tau|$ is even since a physical state must have a specific parity. Now, we evaluate the fermionic partial transpose $\rho^{T_2^f}$ based on Supplementary Eq. (5), and for each term, the operators in subsystem A_2 would be transformed to $\mathcal{R}_2^f(\gamma_{n_1}^{\tau_1} \cdots \gamma_{n_{2l}}^{\tau_{2l}})$. It turns out that the definition in Supplementary Eq. (1) would give us a simple expression for the transformation $\mathcal{R}_2^f$ [7],

$$\mathcal{R}_2^f(\gamma_j) = \mathrm{i}\gamma_j, \quad j \in A_2. \tag{6}$$

Under this fermionic partial transpose, a Gaussian state $\rho_0 = \det[1+e^W]^{-1/2} \exp\left(\frac{1}{4}\sum_{k,l} W_{kl}\gamma_k\gamma_l\right)$ will be transformed to another Gaussian state.

Supplementary Note 2: Determinant Quantum Monte Carlo Methods

In this section, we provide a brief introduction to determinant quantum Monte Carlo (DQMC) methods [8–10]. For our purpose, both the zero-temperature projector scheme and the finite-temperature scheme have been used in the main text.

A. Finite-temperature Scheme

At a finite temperature T, and assuming that the system of interest is in thermodynamic equilibrium, we can analyze it within the framework of the grand canonical ensemble, using the partition function $Z = \mathrm{Tr}\left[e^{-\beta H}\right]$. A generic Hamiltonian H consists of a free-particle term and an interaction term, denoted as $H = H_0 + H_I$. To compute the trace over Fock space, we employ Trotter decomposition and Hubbard-Stratonovich (HS) transformation to factorize the exponential operator $e^{-\beta H}$ into a sum of products of Gaussian operators,

$$\begin{aligned} Z &= \mathrm{Tr}\left[e^{-\beta H}\right] = \mathrm{Tr}\left[\left(e^{-\Delta_\tau H}\right)^{L_\tau}\right] \\ &= \mathrm{Tr}\left[e^{-\Delta_\tau H_I} e^{-\Delta_\tau H_0} \cdots e^{-\Delta_\tau H_I} e^{-\Delta_\tau H_0}\right] + O\left(\Delta_\tau^2\right) \\ &\approx \sum_{\{s_{i,l}\}} \mathrm{Tr}\left[\prod_{l=1}^{L_\tau} \left(e^{\mathbf{c}^\dagger V(l)\mathbf{c}} e^{\mathbf{c}^\dagger K \mathbf{c}}\right)\right], \end{aligned} \tag{7}$$

where $L_\tau = \beta/\Delta_\tau$ is the number of time slices, $-\Delta_\tau H_0 = \mathbf{c}^\dagger K \mathbf{c}$ with $\mathbf{c} = (c_1, \dots, c_N)^T$, and we have decoupled the interaction term H_I to fermion bilinears $\mathbf{c}^\dagger V(l)\mathbf{c} = \mathbf{c}^\dagger V[\mathbf{s}(l)]\mathbf{c}$ coupled with spacetime-dependent auxiliary fields $\mathbf{s} = \{s_{i,l}, i \in 1, \dots, N_c; l = 1, \dots, L_\tau\}$. Here, $\mathbf{s}(l)$ includes all the auxiliary fields at time slice l and N_c represents the number of coupling terms, which varies depending on the specific interactions and decoupled channels. For the Hubbard model, we decouple it to the density channel,

$$e^{-\Delta_\tau \frac{U}{2}\sum_i (n_i - 1)^2} = \sum_{\{s_i = \pm 1, \pm 2\}} \left(\prod_i \gamma(s_i) e^{-\mathrm{i}\sqrt{\Delta_\tau U/2}\,\eta(s_i)}\right) e^{\mathrm{i}\sqrt{\Delta_\tau U/2}\sum_i \eta(s_i) n_i}, \tag{8}$$

where $\gamma(\pm 1) = 1 + \sqrt{6}/3, \gamma(\pm 2) = 1 - \sqrt{6}/3, \eta(\pm 1) = \pm\sqrt{2(3 - \sqrt{6})}$ and $\eta(\pm 2) = \pm\sqrt{2(3 + \sqrt{6})}$. Thus, for the Hubbard model, N_c is the number of sites N. For the spinless t-V model, we decouple it to the hopping channel [11, 12],

$$e^{-\Delta\tau V \sum_{\langle jk \rangle}\left(n_j - \frac{1}{2}\right)\left(n_k - \frac{1}{2}\right)} = \sum_{\{s_{jk} = \pm 1\}} \left(\frac{1}{2} e^{-\frac{V\Delta\tau}{4}}\right) e^{\lambda \sum_{\langle jk \rangle} s_{jk}\left(c_j^\dagger c_k + c_k^\dagger c_j\right)}, \tag{9}$$

where $\cosh\lambda = e^{\frac{V\Delta\tau}{2}}$. Thus, for the t-V model, the subscript i of auxiliary fields $s_{i,l}$ denotes various nearest neighboring (NN) bonds $\langle jk\rangle$, and N_c represents the number of NN bonds (specifically, for bipartite lattices $N_c = Nz/2$ with z being the coordination number). The trace of products of Gaussian operators over the fermionic Fock space in the last line of Supplementary Eq. (7) can be expressed as a determinant,

$$Z = \sum_{\mathbf{s}} \omega_{\mathbf{s}} = \sum_{\mathbf{s}} \alpha_{\mathbf{s}} \det\left[I + \prod_{l=L_\tau,\cdots,1} B_{\mathbf{s}}(l)\right] \text{ with } B_{\mathbf{s}}(l) = e^{V(l)} e^{K}, \tag{10}$$

where $\alpha_{\mathbf{s}}$ denotes the coefficients before the exponential operators. For the Hubbard model, $\alpha_{\mathbf{s}} = \left(\prod_{i,l}\gamma(s_{i,l}) e^{-\mathrm{i}\sqrt{\Delta_\tau U/2}\eta(s_{i,l})}\right)$, while for the t-V model, $\alpha_{\mathbf{s}} = \prod_{\langle jk\rangle,l}\left(\frac{1}{2}e^{-\frac{V\Delta\tau}{4}}\right)$. In addition, the expectation of arbitrary operator O can also be decomposed into a sum over auxiliary fields,

$$\langle O\rangle = \frac{\mathrm{Tr}\left[\mathrm{e}^{-\beta H} O\right]}{\mathrm{Tr}\left[\mathrm{e}^{-\beta H}\right]} = \sum_{\mathbf{s}} P_{\mathbf{s}} \langle O\rangle_{\mathbf{s}} + O\left(\Delta_\tau^2\right) \text{ with } P_{\mathbf{s}} = \frac{\omega_{\mathbf{s}}}{\sum_{\mathbf{s}}\omega_{\mathbf{s}}}. \tag{11}$$

Here, the expectation of O with respect to a specific configuration of auxiliary field is given by

$$\langle O\rangle_{\mathbf{s}} = \frac{\mathrm{Tr}\left[U_{\mathbf{s}}(\beta,\tau) O U_{\mathbf{s}}(\tau,0)\right]}{\mathrm{Tr}\, U_{\mathbf{s}}(\beta,0)} \text{ with } U_{\mathbf{s}}(\tau_2 = l_2\Delta_\tau, \tau_1 = l_1\Delta_\tau) = \prod_{l=l_1+1}^{l_2}\left(e^{\mathbf{c}^\dagger V(l)\mathbf{c}} e^{\mathbf{c}^\dagger K\mathbf{c}}\right). \tag{12}$$

For instance, the most elementary observable, namely the equal-time Green's function, can be calculated via $G_{\mathbf{s},ij}(\tau,\tau) = \langle c_i c_j^\dagger\rangle_{\mathbf{s}} = (1 + B_{\mathbf{s}}(\tau,0)B_{\mathbf{s}}(\beta,\tau))^{-1}_{ij}$ where $B_{\mathbf{s}}(\tau_2 = l_2\Delta_\tau, \tau_1 = l_1\Delta_\tau) = \prod_{l=l_1+1}^{l_2}\left(e^{V(l)}e^{K}\right)$ is the matrix correspondence of $U_{\mathbf{s}}(\tau_2,\tau_1)$.

In practice, the weighted sums in Supplementary Eqs. (10) and (11) are conducted by using Monte-Carlo importance sampling. Specifically, we adopt a local update scheme where the auxiliary fields are updated sequentially, and the acceptance ratio is determined by the Metropolis algorithm. Let us consider an update which is proposed to happen at time slice l and site i, i.e., $s_{i,l} \to s'_{i,l}$, which leads to a change of the determinant weight $\det[I + \prod_l B_{\mathbf{s}}(l)]$: $e^{V'(l)} \equiv (I+\Delta)e^{V(l)}$. For simplicity, we assume that Δ is a diagonal matrix with k nonzero elements. Whether the update is accepted or not is determined by the Metropolis acceptance probability $P_{\mathrm{acc}} = \min\left\{1, \frac{\omega_{\mathbf{s}'}}{\omega_{\mathbf{s}}}\right\}$, where the update ratio can be calculated as

$$\begin{aligned}\frac{\omega_{\mathbf{s}'}}{\omega_{\mathbf{s}}} &= \frac{\alpha_{\mathbf{s}'}}{\alpha_{\mathbf{s}}}\frac{\det\left[I + B_{\mathbf{s}}(\beta,\tau)(I+\Delta)B_{\mathbf{s}}(\tau,0)\right]}{\det\left[I + B_{\mathbf{s}}(\beta,0)\right]} \\ &= \frac{\alpha_{\mathbf{s}'}}{\alpha_{\mathbf{s}}}\frac{\det G_{\mathbf{s}}(\tau,\tau)}{\det G_{\mathbf{s}'}(\tau,\tau)} = \frac{\alpha_{\mathbf{s}'}}{\alpha_{\mathbf{s}}}\det\left[I_k + VU\right]\end{aligned} \tag{13}$$

Here, we have used the fast update formula for the Green's function matrix,

$$G_{\mathbf{s}'}(\tau,\tau) = G_{\mathbf{s}}(\tau,\tau) - G_{\mathbf{s}}(\tau,\tau)U(I_k + VU)^{-1}V, \tag{14}$$

where we define $U \equiv P_{N\times k}D$ and $V \equiv P_{k\times N}(I - G_{s_i}(\tau,\tau))$. They are rectangular matrices that satisfy $\Delta(I - G_{\mathbf{s}}) \equiv UV$. The projection matrices $P_{N\times k}$ and $P_{k\times N}$ are cropped from the identity matrix $I = I_N$, with columns or rows corresponding to the non-zero entries of Δ. If the update is accepted, then we use the above formula to update $G_{\mathbf{s}}$. After finishing the updates of all sites at time slice l, we move to the next time slice by propagating the Green's function matrix using

$$\begin{aligned}G_{\mathbf{s}}(\tau+\Delta_\tau, \tau+\Delta_\tau) &= B_{\mathbf{s}}(l+1)\, G_{\mathbf{s}}(\tau,\tau) B_{\mathbf{s}}(l+1)^{-1}, \\ G_{\mathbf{s}}(\tau-\Delta_\tau, \tau-\Delta_\tau) &= B_{\mathbf{s}}(l)^{-1}\, G_{\mathbf{s}}(\tau,\tau) B_{\mathbf{s}}(l).\end{aligned} \tag{15}$$

At each time slice, we perform one measurement of the observables of interest, which can be expressed by the Green's functions by means of Wick's theorem. For example, the density-density correlation function can be calculated as $\langle n_i n_j\rangle_{\mathbf{s}} = \langle c_i^\dagger c_i\rangle_{\mathbf{s}}\langle c_j^\dagger c_j\rangle_{\mathbf{s}} + \langle c_i^\dagger c_j\rangle_{\mathbf{s}}\langle c_i c_j^\dagger\rangle_{\mathbf{s}}$. After several updates across different time slices, the Green's function matrix requires numerical stabilization based on matrix decomposition. For instance, to stably calculate $G_{\mathbf{s}}(\tau,\tau)$, we can first perform singular value decompositions: $B_{\mathbf{s}}(\beta,\tau)^\dagger = U_L D_L V_L$ and $B_{\mathbf{s}}(\tau,0) = U_R D_R V_R$, and then calculate $G_{\mathbf{s}}(\tau,\tau)$ using

$$G_{\mathbf{s}}(\tau,\tau) = U_L D_{L+}^{-1}\left(D_{R+}^{-1}U_R^\dagger U_L D_{L+}^{-1} + D_{R-}V_R V_L^\dagger D_{L-}\right)^{-1} D_{R+}^{-1}U_R^\dagger, \tag{16}$$

where $D_{L/R,+} = \max(D_{L/R}, 1)$ and $D_{L/R,-} = \min(D_{L/R}, 1)$.

B. Zero-temperature Projector Scheme

In the projector DQMC scheme, the ground-state wavefunction of interest is calculated by projecting from a trial wavefunction $|\Psi_T\rangle$. It is important to note that the trial wavefunction should not be orthogonal to the true ground state $|\psi_0\rangle$ so that it is possible to obtain $|\psi_0\rangle = \lim_{\Theta\to\infty} e^{-\Theta H}|\Psi_T\rangle$ for a sufficiently long projection length Θ. Analogous to the finite temperature case, the modulus of the ground state (which plays the role of the "partition function") and the ground-state expectation of some observable O are written as summations over auxiliary fields after doing Trotter decomposition and HS transformation. The magnitude of the ground state is given by

$$\langle \Psi_0 \mid \Psi_0\rangle = \left\langle \Psi_T \left| e^{-2\Theta H} \right| \Psi_T \right\rangle \approx \sum_{\mathbf{s}} \omega_{\mathbf{s}} = \sum_{\mathbf{s}} \alpha_{\mathbf{s}} \det\left[P^\dagger B_{\mathbf{s}}(2\Theta,0)P\right], \tag{17}$$

where P is the coefficient matrix of the trial state determined by $|\Psi_T\rangle = \prod_{n=1}^{N_p} \left(\mathbf{c}^\dagger P\right)_n |0\rangle$, with N_p representing the number of occupied single-particle states. Here one can also see that 2Θ plays a similar role to β in the finite-temperature case. The observable expectation is given by

$$\langle O\rangle = \sum_{\mathbf{s}} P_{\mathbf{s}} \langle O\rangle_{\mathbf{s}} = \frac{\sum_{\mathbf{s}} \omega_{\mathbf{s}} \langle O\rangle_{\mathbf{s}}}{\sum_{\mathbf{s}} \omega_{\mathbf{s}}} \text{ with } \langle O\rangle_{\mathbf{s}} = \frac{\langle \Psi_T \left| U_{\mathbf{s}}(2\Theta,\tau)\, O U_{\mathbf{s}}(\tau,0) \right| \Psi_T\rangle}{\langle \Psi_T \left| e^{-2\Theta H} \right| \Psi_T\rangle}. \tag{18}$$

For instance, the equal-time Green's function can be calculated as $G_{\mathbf{s}}(\tau,\tau) = I - R(\tau)(L(\tau)R(\tau))^{-1}L(\tau)$ with $R(\tau) = B_{\mathbf{s}}(\tau,0)P$ an $N\times N_p$ matrix, and $L(\tau) = P^\dagger B_{\mathbf{s}}(2\Theta,\tau)$ an $N_p \times N$ matrix.

In practice, we use a similar Monte-Carlo importance sampling method as the finite-temperature case to evaluate the weighted sums in Supplementary Eqs. (17) and (18). Instead of updating the Green's function matrix G, it is more efficient to update the matrices R and $(LR)^{-1}$ using

$$R' = (I+\Delta)R \text{ and } \left[(LR)^{-1}\right]' = (LR)^{-1} - (LR)^{-1}U(I_k + VU)^{-1}V, \tag{19}$$

where $U \equiv L\Delta P_{N\times k}$ and $V \equiv P_{k\times N} R(LR)^{-1}$. The acceptance ratio is given by

$$\frac{\omega_{\mathbf{s}'}}{\omega_{\mathbf{s}}} = \frac{\alpha_{\mathbf{s}'} \det\left[(LR)'\right]}{\alpha_{\mathbf{s}} \det[LR]} = \frac{\alpha_{\mathbf{s}'}}{\alpha_{\mathbf{s}}} \det\left[I_k + VU\right]. \tag{20}$$

The propagation of $L(\tau)$ and $R(\tau)$ is straightforward using

$$L(\tau+\Delta_\tau) = L(\tau)B_{\mathbf{s}}(l+1)^{-1} \text{ and } R(\tau+\Delta_\tau) = B_{\mathbf{s}}(l+1)R(\tau). \tag{21}$$

As for the measurements, we note that to obtain an accurate representation of the true ground state, it is advisable to only perform measurements around $\tau = \Theta$. Finally, the numerical stabilization of Green's function matrix is much easier in the projector scheme. For example, we can first perform QR decompositions: $L_{N_p\times N} = D_L\,(U_L)_{N_p\times N}$ and $R_{N\times N_p} = (U_R)_{N\times N_p} D_R$, and then calculate $G(\Theta,\Theta)$ using

$$G = 1 - R(LR)^{-1}L = 1 - U_R\,(U_L U_R)^{-1}\,U_L. \tag{22}$$

According to the above formula, we can update the U_R, U_L and $(U_L U_R)^{-1}$ matrices instead of R, L and $(LR)^{-1}$, respectively, which is more stable and utilized in practice.

Supplementary Note 3: DQMC implementation of fermionic partial transpose

In this section, we provide a comprehensive discussion of the DQMC implementation of fermionic partial transpose. For completeness, we begin with the formulation of DQMC in the Majorana basis and then transition back to the complex fermion basis.

A. Decomposition of interacting density matrix

Before starting the discussion, we first introduce the so-called *product rule of Gaussian states*, which means that the product of two Gaussian states is still a Gaussian state. This property can be proved using the Baker-Campbell-Hausdorff formula,

$$e^{\hat{W}''} \equiv e^{\hat{W}} e^{\hat{W}'} = e^{\hat{W}+\hat{W}'+\frac{1}{2}[\hat{W},\hat{W}']+\frac{1}{12}([\hat{W},[\hat{W},\hat{W}']]+[\hat{W}',[\hat{W},\hat{W}']])+\cdots}, \tag{23}$$

and the following commuting relations

$$[\gamma_l\gamma_n, \gamma_j\gamma_k] = -2\gamma_l\gamma_j\delta_{kn} + 2\gamma_l\gamma_k\delta_{jn} + 2\gamma_n\gamma_j\delta_{lk} - 2\gamma_n\gamma_k\delta_{lj}, \tag{24a}$$

$$\left[c_l^\dagger c_n, c_j^\dagger c_k\right] = c_l^\dagger c_k\delta_{nk} - c_j^\dagger c_n\delta_{lk}. \tag{24b}$$

As a result, provided that both $e^{\hat{W}}$ and $e^{\hat{W}'}$ are Gaussian states (up to normalization), we can assert that $\hat{W}''$ is a fermionic quadratic, thus $e^{\hat{W}''}$ is a Gaussian state (up to normalization).

In the framework of DQMC, the partition function is given by $Z = \sum_{\mathbf{s}} \mathrm{Tr}[\prod_{l=1}^{L_\tau} e^{\mathbf{c}^\dagger K_l[\mathbf{s}]\mathbf{c}}]$, where K_l combines the K and $V(l)$ in Supplementary Eq. (7) by means of the product rule of Gaussian states. We then convert to the Majorana basis by rewriting the decoupled Hamiltonian as $\mathbf{c}^\dagger K_l[\mathbf{s}]\mathbf{c} = \boldsymbol{\gamma}^T h_l[\mathbf{s}]\boldsymbol{\gamma}/4$, where h_l is a $2N\times 2N$ antisymmetric matrix satisfying the antisymmetric condition $h_l = -h_l^T$ and $\boldsymbol{\gamma} = (\gamma_1, \ldots, \gamma_{2N})$. It is worth noting that this form can encompass terms beyond particle-number conserving terms, such as pairing terms [13–15]. The partition function in the Majorana basis is given by [11, 14, 15]

$$\begin{aligned} Z &= \sum_{\mathbf{s}} \mathrm{Tr}\left[e^{\frac{1}{4}\boldsymbol{\gamma}^T h_{L_\tau}[\mathbf{s}]\boldsymbol{\gamma}} \cdots e^{\frac{1}{4}\boldsymbol{\gamma}^T h_l[\mathbf{s}]\boldsymbol{\gamma}} \cdots e^{\frac{1}{4}\boldsymbol{\gamma}^T h_1[\mathbf{s}]\boldsymbol{\gamma}}\right] = \sum_{\mathbf{s}} \mathrm{Tr}\left[e^{\frac{1}{4}\boldsymbol{\gamma}^T W_{\mathbf{s}}\boldsymbol{\gamma}}\right] \\ &= \sum_{\mathbf{s}} \pm \det\left[I + e^{h_{L_\tau}} \cdots e^{h_l} \cdots e^{h_1}\right]^{1/2} = \sum_{\mathbf{s}} \pm \det\left[I + e^{W_{\mathbf{s}}}\right]^{1/2}, \end{aligned} \tag{25}$$

where we have introduce $W_{\mathbf{s}}$ by further utilizing the product rule of Gaussian states. Therefore, the density matrix of an interacting system can be decomposed to a sum of Gaussian states [16]

$$\rho = \sum_{\mathbf{s}} p_{\mathbf{s}}\rho_{\mathbf{s}}, \quad \rho_{\boldsymbol{s}} = \det\left[I + e^{W_{\mathbf{s}}}\right]^{-1/2} e^{\frac{1}{4}\boldsymbol{\gamma}^T W_{\mathbf{s}}\boldsymbol{\gamma}}. \tag{26}$$

B. Fermionic partial transpose of a single Gaussian state

First of all, we discuss the fermionic partial transpose of a single Gaussian state, i.e. the $\rho_{\mathbf{s}}$ in Supplementary Eq. (26). We may abbreviate the subscript $\mathbf{s}$. These results are related to the Supplementary Eqs. (2) and (3), and also the statements around them in the main text.

The "Green's function" in the Majorana basis is defined as $\Gamma_{\mathbf{s},kl} = \langle[\gamma_k, \gamma_l]\rangle_{\mathbf{s}}/2 = \langle \frac{1}{4}\gamma^T O^{kl}\gamma\rangle_{\mathbf{s}}$ with matrix $O^{kl}_{ij} = 2\delta_{ik}\delta_{jl} - 2\delta_{il}\delta_{jk}$. Γ is also called covariance matrix which characterizes a Gaussian state with relation $\tanh(-W/2) = \Gamma$ [6] or inverse relation $W = \ln\left[(I+\Gamma)^{-1}(I-\Gamma)\right]$. To prove these relations, we treat $\frac{1}{4}\boldsymbol{\gamma}^T O^{kl}\boldsymbol{\gamma}$ as a generic observable and calculate its expectation as

$$\begin{aligned} \Gamma_{\mathbf{s},kl} &= \langle\frac{1}{4}\boldsymbol{\gamma}^T O^{kl}\boldsymbol{\gamma}\rangle_{\mathbf{s}} = \mathrm{Tr}\left[\rho_{\mathbf{s}}\frac{1}{4}\boldsymbol{\gamma}^T O^{kl}\boldsymbol{\gamma}\right] \\ &= \frac{\partial}{\partial\eta}\ln\mathrm{Tr}\left[e^{\frac{1}{4}\boldsymbol{\gamma}^T W_{\mathbf{s}}\boldsymbol{\gamma}} e^{\frac{1}{4}\eta\boldsymbol{\gamma}^T O^{kl}\boldsymbol{\gamma}}\right]\Bigg|_{\eta=0} = \frac{1}{2}\frac{\partial}{\partial\eta}\mathrm{Tr}\ln\left[I + e^{W_{\mathbf{s}}}e^{\eta O^{kl}}\right]\Bigg|_{\eta=0} \\ &= \frac{1}{2}\mathrm{Tr}\left[(I+e^{W_{\mathbf{s}}})^{-1}e^{W_{\mathbf{s}}}O^{kl}\right] = \frac{1}{2}\left[(I+e^{W_{\mathbf{s}}})^{-1}e^{W_{\mathbf{s}}}\right]_{ji} O^{kl}_{ij} \\ &= \left[(I+e^{W_{\mathbf{s}}})^{-1}e^{W_{\mathbf{s}}}\right]_{lk} - \left[(I+e^{W_{\mathbf{s}}})^{-1}e^{W_{\mathbf{s}}}\right]_{kl}. \end{aligned} \tag{27}$$

Based on the above relation, we can prove the relation between Γ and W,

$$\begin{aligned} \Gamma &= -(I+e^W)^{-1}e^W + e^{-W}(I+e^{-W})^{-1} \\ &= -\left(e^{W/2} - e^{-W/2}\right)\left(e^{W/2} + e^{-W/2}\right)^{-1} = \tanh\left(-W/2\right). \end{aligned} \tag{28}$$

This relation is of importance because it indicates that a covariance matrix can be used to describe a Gaussian state equivalently. We note that the above proof assumes that the trace in Supplementary Eq. (25) is without sign ambiguity. One can find a more general proof in Supplementary Ref. [17].

The "partial transpose of the Green's function" in Majorana basis is quite straightforward via Supplementary Eq. (6),

$$\Gamma^{T_2^f} = \begin{pmatrix} \Gamma^{11} & \mathrm{i}\Gamma^{12} \\ \mathrm{i}\Gamma^{21} & -\Gamma^{22} \end{pmatrix}, \tag{29}$$

where $\Gamma^{bb'}$ represents the block consisting of matrix elements with rows belonging to subsystem A_b and columns belonging to subsystem $A_{b'}$. At the moment, our understanding is limited to the fact that $\Gamma^{T_2^f}$ naturally represents a new Gaussian state, which can generally be expanded using Supplementary Eq. (5):

$$\rho' = \sum_{\kappa,\tau} w'_{\kappa,\tau} \gamma_{m_1}^{\kappa_1} \cdots \gamma_{m_{2k}}^{\kappa_{2k}} \gamma_{n_1}^{\tau_1} \cdots \gamma_{n_{2l}}^{\tau_{2l}}. \tag{30}$$

We can further use Wick's theorem for Majorana monomials (products of $2l$ Majorana operators with index different from each other) [6],

$$\mathrm{Tr}\left(\rho \gamma_{n_1} \gamma_{n_2} \cdots \gamma_{n_{2l}}\right) = \sum_{\pi} \mathrm{sgn}(\pi) \prod_{k=1}^{l} \Gamma_{n_{\pi(2k-1)}, n_{\pi(2k)}}, \tag{31}$$

to identify the new Gaussian state ρ'. Here, ρ is the Gaussian state associated with Γ, and π is a permutation representing different pairs of Majorana operators. On the one hand, we can take ρ' as the Majorana monomial

$$\mathrm{Tr}\left(\rho\rho'\right) = \sum_{\kappa,\tau} w'_{\kappa,\tau} \mathrm{Tr}\left(\rho \gamma_{m_1}^{\kappa_1} \cdots \gamma_{m_{2k}}^{\kappa_{2k}} \gamma_{n_1}^{\tau_1} \cdots \gamma_{n_{2l}}^{\tau_{2l}}\right). \tag{32}$$

On the other hand, we can also use ρ' as the Gaussian state to expand ρ

$$\begin{aligned} \mathrm{Tr}(\rho'\rho) &= \sum_{\kappa,\tau} w_{\kappa,\tau} \mathrm{Tr}\left(\rho' \gamma_{m_1}^{\kappa_1} \cdots \gamma_{m_{2k}}^{\kappa_{2k}} \gamma_{n_1}^{\tau_1} \cdots \gamma_{n_{2l}}^{\tau_{2l}}\right) = \sum_{\kappa,\tau} w_{\kappa,\tau} \sum_{\pi} \mathrm{sgn}(\pi) \prod_{p=1}^{k+l} (\Gamma^{T_2^f})_{\pi(2p-1),\pi(2p)} \\ &= \sum_{\kappa,\tau} w_{\kappa,\tau} \mathrm{i}^{|\tau|} \sum_{\pi} \mathrm{sgn}(\pi) \prod_{p=1}^{k+l} \Gamma_{\pi(2p-1),\pi(2p)} = \sum_{\kappa,\tau} w_{\kappa,\tau} \mathrm{i}^{|\tau|} \mathrm{Tr}\left(\rho \gamma_{m_1}^{\kappa_1} \cdots \gamma_{m_{2k}}^{\kappa_{2k}} \gamma_{n_1}^{\tau_1} \cdots \gamma_{n_{2l}}^{\tau_{2l}}\right). \end{aligned} \tag{33}$$

Upon comparing the two equations above, we observe that the ρ' linked with $\Gamma^{T_2^f}$ is in fact the partially transposed Gaussian state $\rho^{T_2^f}$.

In short, for each Gaussian state $\rho_{\mathbf{s}}$ associated with a specific configuration of the auxiliary field, its partial transpose can be expressed via the partial transpose of Green's function in Supplementary Eq. (29), i.e., $\rho_{\mathbf{s}}^{T_2^f} = \rho_0[\Gamma_{\mathbf{s}}^{T_2^f}]$ with $\rho_0[\Gamma] \sim e^{\boldsymbol{\gamma}^\dagger W \boldsymbol{\gamma}}$ and $\tanh(-W/2) = \Gamma$.

C. Fermionic partial transpose of interacting density matrix

Finally, by partially transposing the auxiliary-field-dependent Gaussian states in Supplementary Eq. (26) separately, we obtain the following weighted sum formulation of the partially transposed density matrix:

$$\rho^{T_2^f} = \sum_{\mathbf{s}} p_{\mathbf{s}} \rho_{\mathbf{s}}^{T_2^f}, \quad \rho_{\mathbf{s}}^{T_2^f} = \det\left[I + e^{W_{\mathbf{s}}^{T_2^f}}\right]^{-1/2} e^{\frac{1}{4}\boldsymbol{\gamma}^T W_{\mathbf{s}}^{T_2^f} \boldsymbol{\gamma}}, \tag{34}$$

where $W_{\mathbf{s}}^{T_2^f} = \ln[(I + \Gamma_{\mathbf{s}}^{T_2^f})^{-1}(I - \Gamma_{\mathbf{s}}^{T_2^f})]$. This formula can be re-expressed in the complex fermion basis, which is more convenient for practical calculations

$$\rho^{T_2^f} = \sum_{\mathbf{s}} p_{\mathbf{s}} \rho_{\mathbf{s}}^{T_2^f}, \quad \rho_{\mathbf{s}}^{T_2^f} = \det\left[G_{\mathbf{s}}^{T_2^f}\right] \exp\left\{\mathbf{c}^\dagger \ln\left[\left(G_{\mathbf{s}}^{T_2^f}\right)^{-1} - I\right] \mathbf{c}\right\}, \tag{35}$$

where

$$G^{T_2^f} = \begin{pmatrix} G^{11} & \mathrm{i}G^{12} \\ \mathrm{i}G^{21} & I - G^{22} \end{pmatrix}. \tag{36}$$

Supplementary Note 4: Additional results of the Rényi negativity

For a more direct comprehension of the distinction between Rényi negativity (RN) and Rényi entropy, one can see Supplementary Fig. 1. In this figure, we compare the Rényi entropy S_2, the Rényi negativity $\mathcal{E}_2$, and the Rényi negativity ratio (RNR) R_2 for a half-filled Hubbard chain. The $S_2 - L_{A_1}$ curve in Supplementary Fig. 1(a) resembles the findings of previous studies (e.g., see Supplementary Ref. [18]). As the temperature rises, the Rényi entropy S_2 experiences a quantum-classical transition, shifting from an area law (inclusive of a logarithmic correction) at zero-temperature to a volume law $S_2 = L_{A_1} \log 4$ in the high-temperature limit. At finite temperatures, while the $S_2 - L_{A_1}$ curve exhibits asymmetry about $L_{A_1} = L/2$, the Rényi negativity (see Supplementary Fig. 1(b)) maintains an arc-like structure. Moreover, the two endpoints of the $\mathcal{E}_2 - L_{A_1}$ curve correspond to the thermal entropy $S_2^{\mathrm{th}} = S_2(L_{A_1} = L) = \mathcal{E}_2(L_{A_1} = 0) = \mathcal{E}_2(L_{A_1} = L) = -\ln(\mathrm{Tr}[\rho^2])$. Upon subtracting the thermal entropy from $\mathcal{E}_2$, the negativity ratio R_2 (see Supplementary Fig. 1(c)) emerges as a more capable indicator of finite-temperature entanglement, given its monotonic decrease with rising temperature. In the high-temperature limit, the $R_2 - L_{A_1}$ curve becomes flat for bulk L_{A_1} values. This plateau can be well described by the formula $\mathcal{E}(LT \gg 1) = \frac{1}{2}\left[\ln\left|\frac{\beta}{\pi}\sinh\left(\frac{\pi L_{A_1}}{\beta}\right)\right| - \frac{\pi L_{A_1}}{\beta}\right] + O\left(\mathrm{e}^{-\pi LT}\right)$ obtained by conformal field theory [3] and reflects the area law.

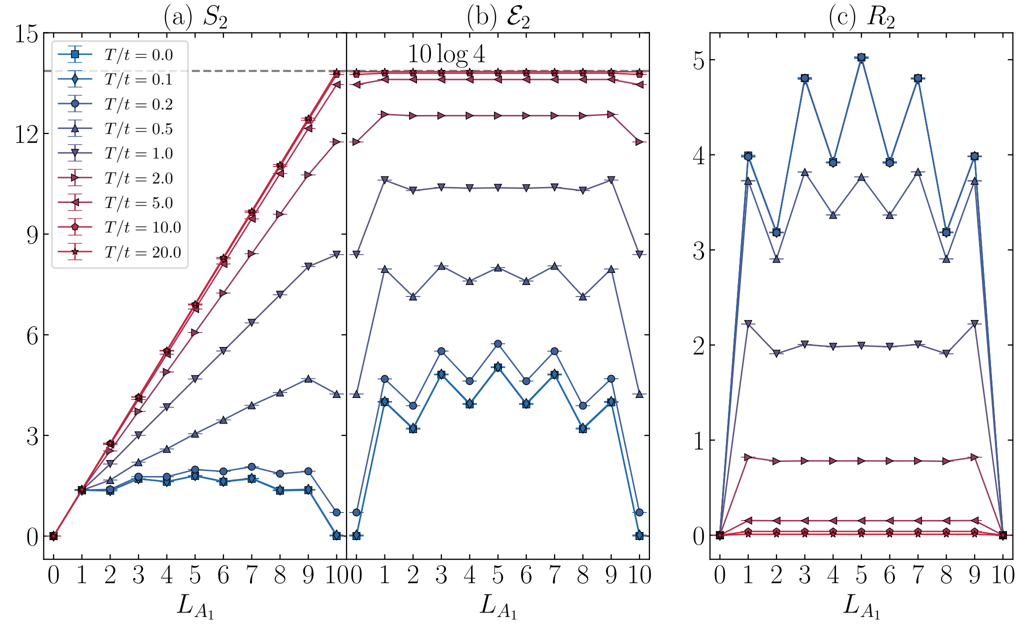

Supplementary Fig. 1. **Comparision of different entanglement measurements for a half-filled Hubbard chain.** The comparison of the Rényi entanglement entropy S_2, the Rényi negativity $\mathcal{E}_2$, and the Rényi negativity ratio R_2 as functions of the subsystem size L_{A_1} is presented for a half-filled Hubbard chain with a length of $L = 10(= L_{A_1} + L_{A_2})$ at various temperatures. The dashed line signifies the thermal entropy in the limit as T approaches infinity, represented by $S_2^{\mathrm{th}}(T \to \infty) = L_{A_1} \log 4$ [18]. The error bars represent the standard errors from Monte Carlo sampling.

As shown in Supplementary Fig. 2, the super-area-law feature near the finite-temperature critical point of the t-V model is evident for two different values of V, a different lattice, and a different bipartition geometry. Figs. 2(a) and (b) show the cases with interaction strengths $V/t = 1.0$ and $V/t = 0.0$ (free fermion case), respectively. The bipartition geometry remains the same as that in Fig. 3 of the main text and is indicated by the right inset of Supplementary Fig. 2(a). In the $V/t = 1.0$ case, the peak of R_2/L is located at $T = 0.833t \approx 2T_c$, aligning with prior findings of $T_c/t \approx 0.4 \pm 0.1$ obtained by DQMC [19]. In the free fermion case, since $T = 0$ is the critical point, the $L \ln L$ scaling is observed at low temperatures, likely associated with the entanglement entropy of the Fermi surface as previous studies on negativity stated [3, 20]. Surprisingly, this scaling persists to a considerable extent at higher temperatures, indicating a prolonged crossover. Supplementary Fig. 2(c) depicts the scenarios with $V/t = 2.0$ on a honeycomb lattice. The super-area-law feature is still evident, and the maximum of R_2/L is observed at $T = 1.111t \approx 2T_c$, which is slightly higher than the previous result of $T_c/t \approx 0.472$ obtained by continuous-time QMC [21]. Supplementary Fig. 2(d) presents the case with an equal bipartition geometry on a square lattice. Despite the subpar data quality, the super-area-law feature around the same critical point as the quarter bipartition case is also observed.

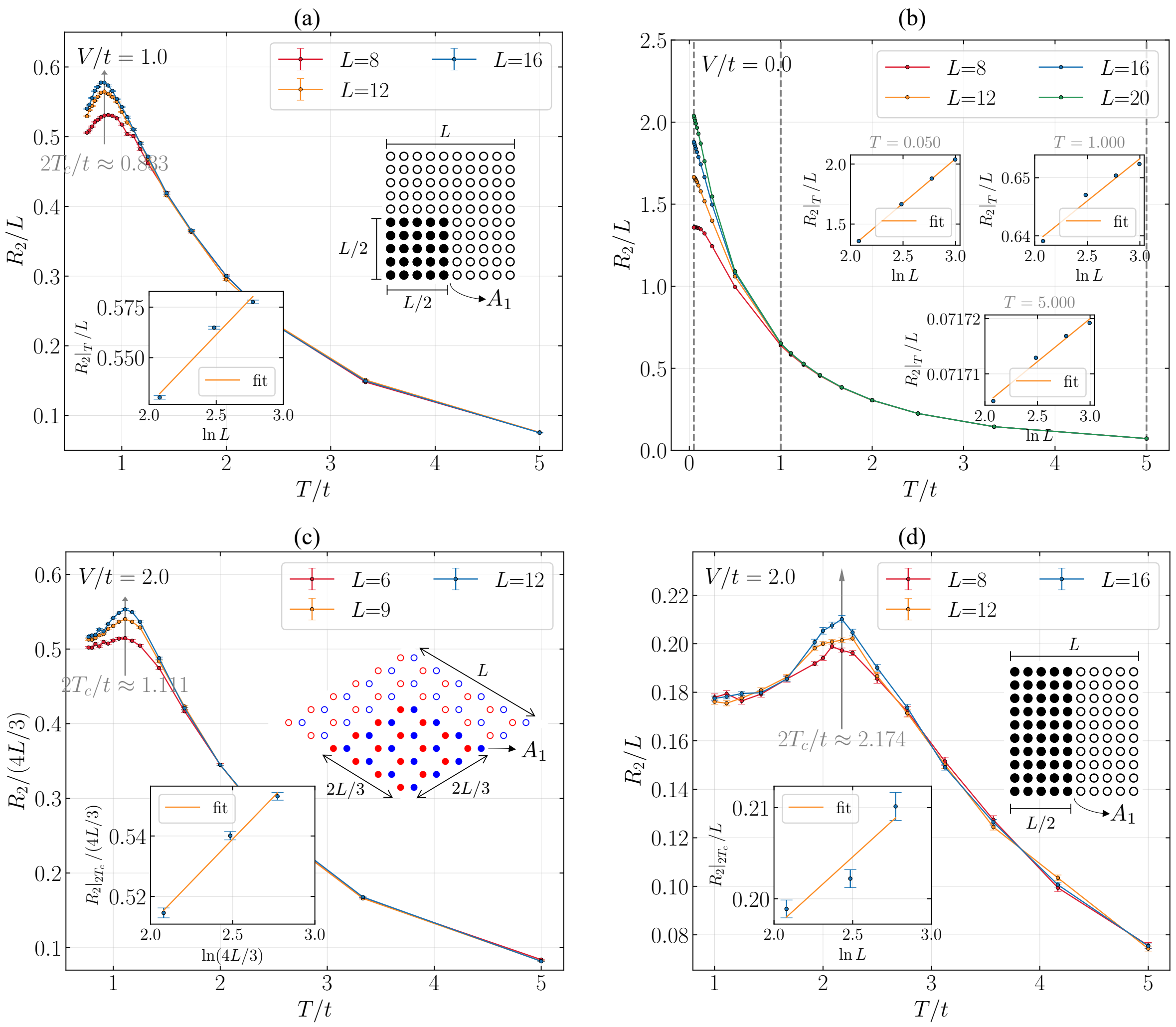

Supplementary Fig. 2. **The beyond-area-law behaviors of the Rényi negativity ratio R_2 for the t-V model in different cases.** The area-law coefficients of the Rényi negativity ratio R_2 as functions of temperature for the t-V model. A super-area-law feature around the finite-temperature critical point is evident for (a) $V/t = 1.0$, (b) $V/t = 0$ (free fermion case), (c) $V/t = 2.0$ on a honeycomb lattice and (d) equal-bipartition geometry. In panels (a) and (b), the bipartition geometry matches that of Fig. 3 in the main text, as indicated by the right inset of (a). The error bars represent the standard errors from Monte Carlo sampling.

Supplementary Note 5: Sign problem of Grover determinant

Using the expression for $\rho_{\mathbf{s}}^{T_2^f}$, we can calculate rank-$n$ Rényi negativity within DQMC framework,

$$e^{-(n-1)\mathcal{E}_n} = \mathrm{Tr}\left[\left(\rho^{T_2^f}\right)^n\right] = \sum_{\mathbf{s}_1\ldots\mathbf{s}_n} P_{\mathbf{s}_1}\cdots P_{\mathbf{s}_n}\,\mathrm{Tr}\left[\rho_{\mathbf{s}_1}^{T_2^f}\cdots\rho_{\mathbf{s}_n}^{T_2^f}\right] = \sum_{\mathbf{s}_1\ldots\mathbf{s}_n} P_{\mathbf{s}_1}\cdots P_{\mathbf{s}_n}\det g_x^n = \langle\det g_x^n\rangle\,, \tag{37}$$

where we have defined the so-called *Grover matrix* g_x^n

$$g_x^n = G_{\mathbf{s}_1}^{T_2^f}\cdots G_{\mathbf{s}_n}^{T_2^f}\left[I + \left(G_{\mathbf{s}_1}^{T_2^f}\right)^{-1}\left(I - G_{\mathbf{s}_1}^{T_2^f}\right)\cdots\left(G_{\mathbf{s}_n}^{T_2^f}\right)^{-1}\left(I - G_{\mathbf{s}_n}^{T_2^f}\right)\right] \tag{38}$$

and its determinant $\det g_x^n$ called *Grover determinant*. As an entanglement measurement, $\mathcal{E}_n \geq 0$ so that $0 < \langle\det g_x^n\rangle \leq 1$. It is an interesting question whether for any specific configuration of auxiliary fields $\{\mathbf{s}_1,\ldots,\mathbf{s}_n\}$, we always have $\det g_x^n \geq 0$. Moreover, this condition is necessary for the development of an incremental algorithm [22, 23] that can accurately compute Rényi negativity, as the weights of all incremental processes include a factor of $(\det g_x^n)^{1/N}$. In this section, we prove that the Grover determinant is real and positive for two classes of sign-problem-free models. We note that these conditions are also applicable to the corresponding Grover determinant associated with entanglement entropy, where all the $G^{T_2^f}$ in Supplementary Eq. (38) are replaced by G^{A_2}. We only consider models on bipartite lattices and use the notion $(-)^i$ for staggered phase factor that takes 1 (-1) at sites belonging to sublattice A (B).

A. Sufficient Condition I: $G_{ij}^{\downarrow} = (-)^{i+j}(\delta_{ij} - G_{ji}^{\uparrow *})$

The first class of models includes the half-filled Hubbard model on bipartite lattices. After HS transformation that decouples Hubbard term to density channel as in Supplementary Eq. (8), the spacetime-dependent Hamiltonian for a specific configuration of auxiliary fields is given by

$$H = \sum_i \mathrm{i}C_i(n_{i\uparrow} + n_{i\downarrow} - 1) + \sum_{\langle i,j\rangle} D_{ij}(c_{i\uparrow}^\dagger c_{j\uparrow} + c_{i\downarrow}^\dagger c_{j\downarrow} + \mathrm{h.c.}), \tag{39}$$

where C_i and D_{ij} are real constant factors. Turn to a new basis via a partial particle-hole transformation $\tilde{c}_{i\uparrow} = c_{i\uparrow}, \tilde{c}_{i\downarrow} = (-)^i c_{i\downarrow}^\dagger$, we obtain

$$\tilde{H} = \sum_i \mathrm{i}C_i(\tilde{n}_{i\uparrow} - \tilde{n}_{i\downarrow}) + \sum_{\langle i,j\rangle} D_{ij}(\tilde{c}_{i\uparrow}^\dagger \tilde{c}_{j\uparrow} + \tilde{c}_{i\downarrow}^\dagger \tilde{c}_{j\downarrow} + \mathrm{h.c.}) \tag{40}$$

This Hamiltonian possesses an anti-unitary symmetry $i\sigma_y\mathcal{K}$, where σ_y acts on the spin sector and $\mathcal{K}$ means complex conjugate, so it is sign-problem-free. Since the blocks in spin-up and spin-down sectors are complex conjugate to each other, $\tilde{H}_\uparrow = \tilde{H}_\downarrow$, the two blocks in any eigenvector of Hamiltonian and hence the Green's function are also complex conjugate to each other, $\tilde{G}_{ij}^{\downarrow} \equiv \langle\tilde{c}_{i\downarrow}\tilde{c}_{j\downarrow}^\dagger\rangle = \tilde{G}_{ij}^{\uparrow *} \equiv \langle\tilde{c}_{i\uparrow}\tilde{c}_{j\uparrow}^\dagger\rangle$. Return to the original basis, we find that the Green's functions satisfy the following relation

$$G_{ij}^{\downarrow} = (-)^{i+j}(\delta_{ij} - G_{ji}^{\uparrow *}), \tag{41}$$

which can be rewritten as the following matrix form

$$G^{\downarrow} = U^\dagger\left(I - (G^{\uparrow})^\dagger\right)U \tag{42}$$

with $U_{ij} = \delta_{ij}(-)^i = \delta_{ij}(-)^j$ a diagonal unitary matrix.

The condition in Supplementary Eq. (41) is sufficient for $\det g_x^n \geq 0$. Consider the spin-up block of partially transposed Green's function in Supplementary Eq. (36), which satisfies

$$U^\dagger\left(G^{\uparrow,T_2^f}\right)^\dagger U = VG^{\downarrow,T_2^f}V + I, \text{ with } V = \begin{pmatrix}\mathrm{i}I_1 & \\ & -\mathrm{i}I_2\end{pmatrix}. \tag{43}$$

V is a diagonal unitary matrix satisfying $V^2 = -I$. Reformulate the above relation we have

$$\begin{aligned} G^{\downarrow,T_2^f} &= V^\dagger U^\dagger \left(I - \left(G^{\uparrow,T_2^f}\right)^\dagger\right) UV, \\ I - G^{\uparrow,T_2^f} &= UV \left(G^{\downarrow,T_2^f}\right)^\dagger V^\dagger U^\dagger, \\ 1 - G^{\downarrow,T_2^f} &= V^\dagger U^\dagger \left(G^{\uparrow,T_2^f}\right)^\dagger UV. \end{aligned} \tag{44}$$

Using the above relations, one can prove that $\det g_x^n = \det g_x^{n,\uparrow} \det g_x^{n,\downarrow} \geq 0$ since

$$\begin{aligned} \det g_x^{n,\downarrow} &\equiv \det \left\{ G_{\mathbf{s}_1}^{\downarrow,T_2^f} \cdots G_{\mathbf{s}_n}^{\downarrow,T_2^f} \left[I + \left(G_{\mathbf{s}_1}^{\downarrow,T_2^f}\right)^{-1} \left(I - G_{\mathbf{s}_1}^{\downarrow,T_2^f}\right) \cdots \left(G_{\mathbf{s}_n}^{\downarrow,T_2^f}\right)^{-1} \left(I - G_{\mathbf{s}_n}^{\downarrow,T_2^f}\right) \right] \right\} \\ &= \det \left\{ \left[I + G_{\mathbf{s}_n}^{\uparrow,T_2^f} \left(I - G_{\mathbf{s}_n}^{\uparrow,T_2^f}\right)^{-1} \cdots G_{\mathbf{s}_1}^{\uparrow,T_2^f} \left(I - G_{\mathbf{s}_1}^{\uparrow,T_2^f}\right)^{-1} \right] \left(I - G_{\mathbf{s}_n}^{\uparrow,T_2^f}\right) \cdots \left(I - G_{\mathbf{s}_1}^{\uparrow,T_2^f}\right) \right\}^* \\ &= \det \left\{ G_{\mathbf{s}_1}^{\uparrow,T_2^f} \cdots G_{\mathbf{s}_n}^{\uparrow,T_2^f} \left[I + \left(G_{\mathbf{s}_1}^{\uparrow,T_2^f}\right)^{-1} \left(I - G_{\mathbf{s}_1}^{\uparrow,T_2^f}\right) \cdots \left(G_{\mathbf{s}_n}^{\uparrow,T_2^f}\right)^{-1} \left(I - G_{\mathbf{s}_n}^{\uparrow,T_2^f}\right) \right] \right\}^* \\ &\equiv \left(\det g_x^{n,\uparrow}\right)^* . \end{aligned} \tag{45}$$

B. Sufficient Condition II: $\Gamma_{ij}^{(2)} = (-)^{i+j}\Gamma_{ij}^{(1)*}$

The second class of models, including the spinless t-V model on bipartite lattices, are proved in Majorana basis. For convenience, let us first relabel the Majorana operators by introducing a specie index, i.e., we use $\gamma_i^{(1)}$ and $\gamma_i^{(2)}$ to represent γ_{2i-1} and γ_{2i}, respectively. After HS transformation that decouples NN interaction terms to Majorana hopping channel as in Supplementary Eq. (9), the spacetime-dependent Hamiltonian for a specific configuration of auxiliary fields is given by

$$H = \sum_{\langle ij \rangle} C_{ij} \left(\mathrm{i}\gamma_i^{(1)}\gamma_j^{(1)} + \mathrm{i}\gamma_i^{(2)}\gamma_j^{(2)} \right), \tag{46}$$

where C_i are real constant factors. This Hamiltonian possesses an anti-unitary symmetry $T\mathcal{K}$, where T transforms $\gamma_i^{(1)}$ ($\gamma_i^{(2)}$) to $(-)^i\gamma_i^{(2)}$ ($(-)^i\gamma_i^{(1)}$), so it is sign-problem-free. Turn to a new basis via transformation $\tilde{\gamma}_i^{(1)} = \gamma_i^{(1)}, \tilde{\gamma}_i^{(2)} = (-)^i\gamma_i^{(2)}$, the Hamiltonian becomes

$$\tilde{H} = \sum_{\langle ij \rangle} C_{ij} \left(\mathrm{i}\tilde{\gamma}_i^{(1)}\tilde{\gamma}_j^{(1)} - \mathrm{i}\tilde{\gamma}_i^{(2)}\tilde{\gamma}_j^{(2)} \right). \tag{47}$$

Since the coefficients of $\tilde{\gamma}_i^{(1)}\tilde{\gamma}_j^{(1)}$ and $\tilde{\gamma}_i^{(2)}\tilde{\gamma}_j^{(2)}$ are complex conjugate to each other, using similar arguments as the case of Hubbard model, we have the relation $\tilde{\Gamma}^{(2)} = \tilde{\Gamma}^{(1)*}$. Return to the original basis, we find that Green's functions satisfy the following relation

$$\Gamma_{ij}^{(2)} = (-)^{i+j}\Gamma_{ij}^{(1)*} \text{ or } \Gamma^{(2)} = U^\dagger\Gamma^{(1)*}U. \tag{48}$$

The condition stated in Supplementary Eq. (48) is also sufficient for $\det g_x^n \geq 0$. According to the expression of partially transposed Green's function in Supplementary Eq. (29) and $W^{T_2^f} = \ln[(I+\Gamma^{T_2^f})^{-1}(I-\Gamma^{T_2^f})]$, we can derive the relationships between the two blocks pertaining to two distinct Majorana species sectors:

$$\Gamma^{(2),T_2^f} = J^\dagger U^\dagger \left(\Gamma^{(1)T_2^f}\right)^* UJ \text{ and } W^{(2),T_2^f} = J^\dagger U^\dagger \left(W^{(1),T_2^f}\right)^* UJ \text{ with } J = \begin{pmatrix} I_1 & \\ & -I_2 \end{pmatrix}. \tag{49}$$

Write the Grover determinant in Majorana basis using Supplementary Eq. (34), and it can be easily proved that

$\det g_x^n = \det g_x^{n,(1)} \det g_x^{n,(2)} \geq 0$ since

$$\begin{aligned}
\det g_x^{n,(2)} &= \det\left[I + e^{W_{s_1}^{(2),T_2^f}}\right]^{-1/2} \cdots \det\left[I + e^{W_{s_n}^{(2),T_2^f}}\right]^{-1/2} \det\left[I + e^{W_{s_1}^{(2),T_2^f}} \cdots e^{W_{s_n}^{(2),T_2^f}}\right]^{1/2} \\
&= \det\left[I + e^{W_{s_1}^{(1),T_2^f,*}}\right]^{-1/2} \cdots \det\left[I + e^{W_{s_n}^{(1),T_2^f,*}}\right]^{-1/2} \det\left[I + e^{W_{s_1}^{(1),T_2^f,*}} \cdots e^{W_{s_n}^{(1),T_2^f,*}}\right]^{1/2} \\
&= \det g_x^{n,(1)*}.
\end{aligned} \tag{50}$$

[1] G. Vidal and R. F. Werner, Computable measure of entanglement, Physical Review A **65**, 032314 (2002).
[2] K. Shiozaki, H. Shapourian, K. Gomi, and S. Ryu, Many-body topological invariants for fermionic short-range entangled topological phases protected by antiunitary symmetries, Physical Review B **98**, 035151 (2018).
[3] H. Shapourian and S. Ryu, Finite-temperature entanglement negativity of free fermions, Journal of Statistical Mechanics: Theory and Experiment **2019**, 043106 (2019).
[4] E. Fradkin, Disorder operators and their descendants, Journal of Statistical Physics **167**, 427 (2017).
[5] Z. H. Liu, Y. D. Liao, G. Pan, M. Song, J. Zhao, W. Jiang, C.-M. Jian, Y.-Z. You, F. F. Assaad, Z. Y. Meng, and C. Xu, Disorder operator and R\'enyi entanglement entropy of symmetric mass generation, Physical Review Letters **132**, 156503 (2024).
[6] V. Eisler and Z. Zimborás, On the partial transpose of fermionic Gaussian states, New Journal of Physics **17**, 053048 (2015).
[7] H. Shapourian, K. Shiozaki, and S. Ryu, Partial time-reversal transformation and entanglement negativity in fermionic systems, Physical Review B **95**, 165101 (2017).
[8] R. Blankenbecler, D. J. Scalapino, and R. L. Sugar, Monte Carlo calculations of coupled boson-fermion systems. I, Physical Review D **24**, 2278 (1981).
[9] D. J. Scalapino and R. L. Sugar, Monte Carlo calculations of coupled boson-fermion systems. II, Physical Review B **24**, 4295 (1981).
[10] F. Assaad and H. Evertz, World-line and Determinantal Quantum Monte Carlo Methods for Spins, Phonons and Electrons, in *Computational Many-Particle Physics*, Lecture Notes in Physics, edited by H. Fehske, R. Schneider, and A. Weiße (Springer, Berlin, Heidelberg, 2008) pp. 277–356.
[11] Z.-X. Li, Y.-F. Jiang, and H. Yao, Solving the fermion sign problem in quantum Monte Carlo simulations by Majorana representation, Physical Review B **91**, 241117 (2015).
[12] L. Wang, Y.-H. Liu, M. Iazzi, M. Troyer, and G. Harcos, Split orthogonal group: A guiding principle for sign-problem-free fermionic simulations, Physical Review Letters **115**, 250601 (2015).
[13] G. H. Lang, C. W. Johnson, S. E. Koonin, and W. E. Ormand, Monte Carlo evaluation of path integrals for the nuclear shell model, Physical Review C **48**, 1518 (1993).
[14] I. Klich, A note on the full counting statistics of paired fermions, Journal of Statistical Mechanics: Theory and Experiment **2014**, P11006 (2014).
[15] Z.-C. Wei, Semigroup Approach to the Sign Problem in Quantum Monte Carlo Simulations (2018), arXiv:1712.09412 [cond-mat, physics:hep-lat, physics:math-ph, physics:nucl-th].
[16] T. Grover, Entanglement of interacting fermions in quantum Monte Carlo calculations, Physical Review Letters **111**, 130402 (2013).
[17] M. Fagotti and P. Calabrese, Entanglement entropy of two disjoint blocks in XY chains, Journal of Statistical Mechanics: Theory and Experiment **2010**, P04016 (2010).
[18] P. Broecker and S. Trebst, Rényi entropies of interacting fermions from determinantal quantum Monte Carlo simulations, Journal of Statistical Mechanics: Theory and Experiment **2014**, P08015 (2014).
[19] D. J. Scalapino, R. L. Sugar, and W. D. Toussaint, Monte Carlo study of a two-dimensional spin-polarized fermion lattice gas, Physical Review B **29**, 5253 (1984).
[20] V. Eisler and Z. Zimborás, Entanglement negativity in two-dimensional free lattice models, Physical Review B **93**, 115148 (2016).
[21] S. Hesselmann and S. Wessel, Thermal Ising transitions in the vicinity of two-dimensional quantum critical points, Physical Review B **93**, 155157 (2016).
[22] Y. D. Liao, Controllable Incremental Algorithm for Entanglement Entropy in Quantum Monte Carlo Simulations (2023), arXiv:2307.10602 [cond-mat].
[23] In preparation.
